# Sequencing a piece of history: complete genome sequence of the original *Escherichia coli* strain

**DOI:** 10.1099/mgen.0.000106

**Published:** 2017-03-23

**Authors:** Karl A Dunne, Roy R Chaudhuri, Amanda E Rossiter, Irene Beriotto, Douglas F Browning, Derrick Squire, Adam F Cunningham, Jeffrey A Cole, Nicholas Loman, Ian R Henderson

**Affiliations:** ^1^​ Institute of Microbiology and Infection, University of Birmingham, Birmingham, UK; ^2^​ Department of Molecular Biology and Biotechnology, University of Sheffield, Sheffield, UK

**Keywords:** *Escherichia coli*, Theodor Escherich, genome sequence

## Abstract

In 1885, Theodor Escherich first described the *Bacillus coli commune*, which was subsequently renamed *Escherichia coli*. We report the complete genome sequence of this original strain (NCTC 86). The 5 144 392 bp circular chromosome encodes the genes for 4805 proteins, which include antigens, virulence factors, antimicrobial-resistance factors and secretion systems, of a commensal organism from the pre-antibiotic era. It is located in the *E. coli* A subgroup and is closely related to *E. coli* K-12 MG1655. *E. coli* strain NCTC 86 and the non-pathogenic K-12, C, B and HS strains share a common backbone that is largely co-linear. The exception is a large 2 803 932 bp inversion that spans the replication terminus from *gmhB* to *clpB*. Comparison with *E. coli* K-12 reveals 41 regions of difference (577 351 bp) distributed across the chromosome. For example, and contrary to current dogma, *E. coli* NCTC 86 includes a nine gene *sil* locus that encodes a silver-resistance efflux pump acquired before the current widespread use of silver nanoparticles as an antibacterial agent, possibly resulting from the widespread use of silver utensils and currency in Germany in the 1800s. In summary, phylogenetic comparisons with other *E. coli* strains confirmed that the original strain isolated by Escherich is most closely related to the non-pathogenic commensal strains. It is more distant from the root than the pathogenic organisms *E. coli* 042 and O157 : H7; therefore, it is not an ancestral state for the species.

## Abbreviations

CDS, coding DNA sequence; CPS, capsular polysaccharide; CU, chaperone–usher; ETEC, enterotoxigenic Escherichia coli; LPS, lipopolysaccharide; NCTC, National Collection of Type Cultures; PTS, phosphotransferase system; ROD, region of difference; T1SS, type 1 secretion system; T2SS, type 2 secretion system; T3SS, type 3 secretion system; T5SS, type 5 secretion system; T6SS, type 6 secretion system; UPEC, uropathogenic Escherichia coli.

## Data Summary

Supplementary figures have been deposited in Figshare (DOI:10.6084/m9.figshare.4235762) (https://figshare.com/s/91b570d54d27b0ecf9ea). Supplementary tables are available with the online Supplementary Material. The genome sequence of *Escherichia* *coli* NCTC 86 has been deposited in GenBank with project accession number PRJEB14041 (www.ebi.ac.uk/ena/data/view/PRJEB14041) and in EMBL with accession number LT601384 (https://www.ncbi.nlm.nih.gov/nuccore/LT601384).

## Impact Statement

This paper is an acknowledgment to one of the fathers of modern microbiology, Theodor Escherich. Without his contribution, the shape of the biological field today may have been an extremely different one. To give another layer of depth to the story of its origin, we have sequenced the first isolate of *Escherichia* *coli*. The complete genome sequence reveals the genes encoding the antigens, virulence factors, antimicrobial resistance and secretion systems of a commensal organism from the pre-antibiotic era.

## Introduction

*Escherichia coli* is unsurpassed as a model organism in the field of biology. Its leading role is predicated on its ability to replicate rapidly, to adjust easily to nutritional and environmental changes, and the relative simplicity with which it can be genetically manipulated. The origin of *E. coli* as a model organism began in the early part of the twentieth century with Charles Clifton’s study of oxidation–reduction reactions in *E. coli* K-12 and Felix d’Herelle’s studies on bacteriophage interactions with *E. coli* B [1–3]. However, *E. coli* became widely studied after the work of Tatum and Lederberg on amino acid biosynthesis and exchange of genetic material that lead to the award of a Nobel Prize [3]. Subsequently, many Nobel Prizes have been awarded for various studies using this versatile organism.

The depth of knowledge about the biochemistry of *E. coli* and the non-pathogenic character of laboratory strains has made *E. coli* the workhorse of molecular biology. No other organism is exploited more widely in research laboratories to manipulate DNA and to produce native and mutant proteins for studies in a variety of settings. Indeed, Neidhardt’s eloquent statement exemplifies the diverse role of *E. coli* in research ‘*Although not everyone is mindful of it, all cell biologists have two cells of interest, the one they are studying and E. coli*’ [4]. Therefore, as the biotechnology industry arose out of the molecular and cell biology research laboratories, it was logical for *E. coli* to become the cornerstone of these revolutionary endeavours. Today, many biopharmaceuticals are produced in *E. coli*, and these products impact on the lives of millions of individuals worldwide on a daily basis [5].

Whilst *E. coli* rose to prominence in the twentieth century, the origin of this organism dates back to the late nineteenth century. In about 1885, Theodor Escherich (1857–1911) first described *Bacterium coli commune*, later named *Bacillus coli communis* and eventually *E. coli* in Escherich’s honour after his death [6]. In his initial description, Escherich made several important observations that are widely reported in the modern literature, often without supporting citations. Escherich noted: (i) that the intestinal tracts of infants were sterile at birth, but were colonized by *E. coli* within hours of birth; (ii) that bacterial colonization of the intestine was attributable to the infant's environment; (iii) that *E. coli* was a Gram-negative, rod-shaped motile organism; (iv) that *E. coli* was a dominant member of the microbiota; (v) that *E. coli* produced acid and fermented glucose; and (vi) that *E. coli* adopted a commensal lifestyle in the normal host [7]. Soon after, Escherich and others noted the pyogenic and pathogenic properties of certain *E. coli* strains, although these were predominantly thought to be associated with individuals whose health was compromised in some manner. The association of *E. coli* with urinary tract disease was observed relatively quickly [8], although it would be over 50 years before specific diarrhoeal strains of *E. coli* were identified [9].

When Theodor Escherich first described his bacillus some 125 years ago, he could not possibly have imagined the major impact his discovery would have on subsequent generations of scientists. Even at the time of his death, he could not have known how *E. coli* would influence the study of biological science. Despite the significance of his discovery, the historically important strain he isolated has remained largely unstudied. Here, we present the whole-genome sequence of Escherich’s original isolate and compare this genome with the genomes of other pathogenic and commensal *E. coli*.

## Methods

### Bacterial strain and sequencing

The *E. coli* strain NCTC 86 was isolated in 1885 from a child with no overt signs of diarrhoeal disease [7]. The strain was deposited in the National Collection of Type Cultures (NCTC) in 1919 by the Lister Institute and is officially recognized as Escherich’s original *Bacillus coli commune* [7]. The isolate sequenced here was obtained from the UK Health Protection Agency’s NCTC and is derived from batch 1. DNA was prepared using Qiagen genomic DNA preparation kits according to the manufacturer’s instructions. DNA was fragmented using a Hydroshear (Digilab) using the recommended protocol for 20 kb fragments and further size-selected on a BluePippin instrument (Sage Science) with a 7 kb minimum size cut-off. The library was sequenced on two SMRT Cells using the Pacific Biosciences RS II instrument at the Norwegian Sequencing Centre, Oslo, Norway, using C4-P2 chemistry. The *E. coli* genome was generated by *de novo* assembly using the Pacific Biosciences sequencing data. Reads were assembled using the ‘RS_HGAP_Assembly.3’ pipeline within SMRT Portal V2.2.0. Illumina reads from the same sample were mapped to this draft genome assembly in order to correct remaining indel errors in the assembly using Pilon (www.broadinstitute.org/software/pilon).

### Gene prediction, annotation and comparative analysis

Automated annotation was performed using Prokka v1.8 (PMID: 24642063), which uses Prodigal (PMID: 20211023) for coding sequence prediction, Barrnap (www.vicbioinformatics.com/software.barrnap.shtml) to identify rRNA genes, Aragorn to identify tRNA genes (PMID: 14704338) and Infernal (PMID: 24008419) to identify non-coding RNA genes. To assess the size of the core genome and pan-genome of *E. coli*, a set of 35 complete *E. coli* and *Shigella* genomes was selected. A pan-genome analysis was performed using Roary v3.7.0 (PMID: 26198102) to determine the sizes of the core and pan-genomes. A whole-genome phylogeny was reconstructed from the Roary core genome alignment using RAxML (PMID: 24451623).

### Nucleotide sequence accession number

The annotated genome sequence of *E. coli* NCTC 86 has been deposited in the EMBL database with the accession number LT601384.

## Results and Discussion

### Structure and general features of the *E. coli* NCTC 86 chromosome

**Fig. 1.**
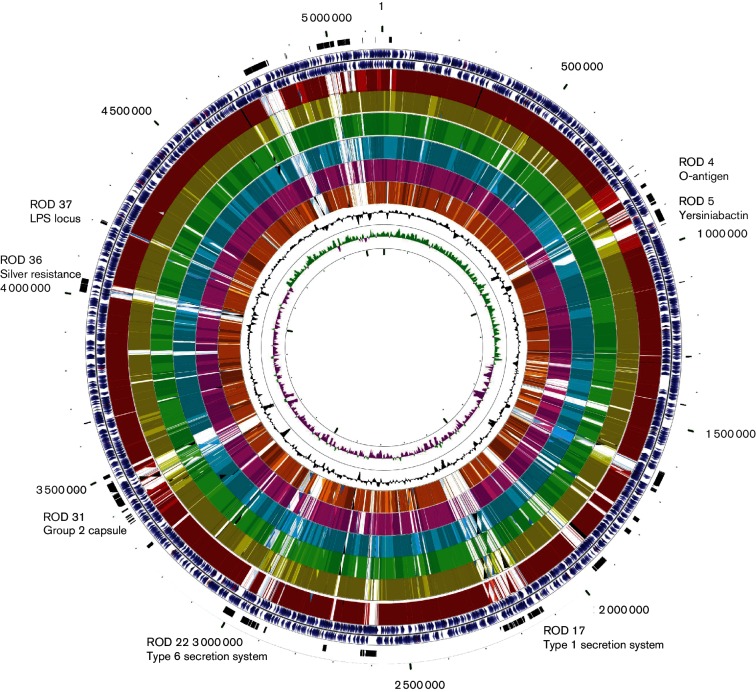
Circular representation of the *E. coli* NCTC 86 chromosome. From the outside to inside, circle 1 shows the sizes in bp. Circle 2 marks the positions of RODs. Circles 3 and 4 show the positions of CDSs transcribed in a clockwise and anticlockwise direction. Circles 5 to 10 show the positions of *E. coli* NCTC 86 genes that have orthologues (by reciprocal fasta analysis) in other *E. coli* strains: K-12 MG1655 (red), E24377A (ETEC; yellow), 042 (ETEC; green), Sakai (0157 : H7; blue), IAI39 (Extraintestinal Pathogenic *E. coli*; pink), CFT073 (UPEC; orange). Circle 11 shows a plot of G+C content. Circle 12 shows a plot of G+C skew.

The *E. coli* NCTC 86 genome consists of a circular chromosome of 5 144 392 bp. The general features of the *E. coli* NCTC 86 chromosome are presented in Table 1. We identified 4805 protein-encoding genes (coding DNA sequences, CDSs) in the chromosome, which included 414 (8.62%)that encoded conserved hypothetical proteins with no known function and 544 (11.32 %) genes associated with mobile elements such as integrases or transposases, or that were phage related. We have identified 41 regions of difference (RODs) in *E. coli* NCTC 86 compared with other sequenced *E. coli* chromosomes (Fig. 1, Table S1, available in the online Supplementary Material). The combined size of these RODs was 577 351 bp (11.22 % of the chromosome). They included 10 prophages distributed across the chromosome (Fig. 1).

**Table 1.** Major features of the genome of *E. coli* NCTC 86 and several archetypal *E. coli* strains

Characteristic	*E. coli* strain					
	NCTC 86	MG1655	O157 : H7	E24377A	042	CFT073
Aetiology	Commensal	Laboratory	EHEC	ETEC	EAEC	UPEC
Size (bp)	5 144 392	4 641 652	5 594 477	5 249 288	5 241 977	5 231 428
Plasmids	None	None	pO157, pOSAK1	pETEC_5, pETEC_6, pETEC_35, pETEC_73, pETEC_74, pETEC_80	pAA	None
Predicted CDSs	4805	4377	5292	4991	4810	5533
G+C mol%	50.6	51.1	50.4	50.6	50.56	50.47
tRNA	69	86	103	63	93	89
rRNA	22	22	22	22	22	22

EAEC, Enteroaggregative *E. coli*; EHEC, enterohaemorrhagic *E. coli*; ETEC, enterotoxigenic *E. coli*.

**Fig. 2.**
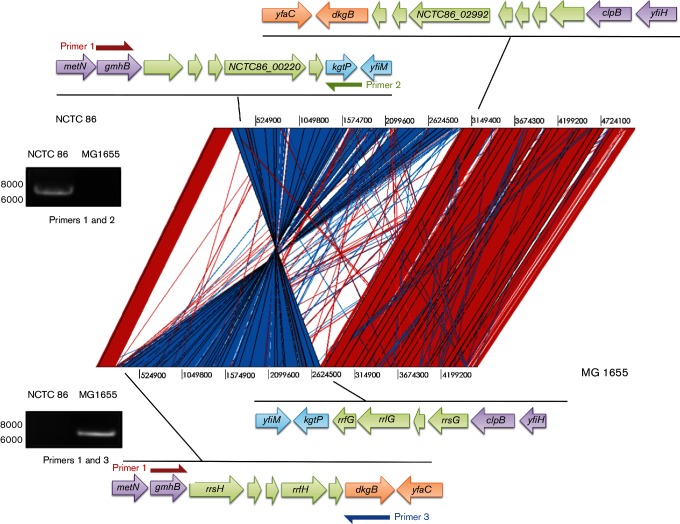
Global comparison between of the chromosomes of *E. coli* NCTC 86 and *E. coli* K-12 MG1655. The red bars between the DNA lines represent individual tblastx matches, with inverted matches coloured blue. Regions where inversion occurs have been expanded, with RNA genes in green, backbone flanking regions in purple, and either side of the inversion in blue and orange. The products arising from PCR amplification of genomic DNA with primers corresponding to *gmhB* (primer 1) and *dkgB* (primer 2) or *kgtP* (primer 3) were separated by agarose gel electrophoresis and are shown in insets; sizes (bp) are shown on the left of the gel images.

Comparison of *E. coli* NCTC 86 with the non-pathogenic *E. coli* K-12, C, B and HS strains revealed that these genomes shared a common backbone that is largely collinear, with the exception of a 2 803 932 bp inversion (Fig. 2). The inversion spans the replication terminus from *gmhB* to *clpB*. The inversion was apparent from long Pacific Biosciences (PacBio) reads and was generated by recombination between the equivalent DNA of the *E. coli* K-12 *rrfH* and *rrsG* RNA genes. PCR was used to confirm this intrachromosomal recombination event. Amplification of *E. coli* K-12 genomic DNA with primers corresponding to regions within *gmhB* and *dkgB* resulted in a product of 6.8 kb. No amplification product could be detected from similar reactions with *E. coli* NCTC 86 genomic DNA (Fig. 2). In contrast, PCR amplification of *E. coli* NCTC 86 genomic DNA with primers corresponding to regions within *gmhB* and *kgtP* resulted in a product of 7.2 kb, while no PCR product was obtained from reactions with the same primers and *E. coli* K-12 DNA (Fig. 2). The size of the PCR products observed in these experiments is consistent with the distance between these genes predicted from the genome sequence data. The absence of an amplification product indicates that the target genes are located distally on the chromosome. Whilst these results confirm the inversion, the functional significance of this recombination event is unknown. This phenomenon has been noted before to occur in laboratory strains, but it was suggested that strains with such inversions rarely survive in the environment [10]. The inversion in *E. coli* NCTC 86 may have arisen due to prolonged culture and storage under laboratory conditions. However, such inversions have been noted in strains more recently isolated from humans [11].

### Phylogeny of *E. coli* NCTC 86

**Fig. 3.**
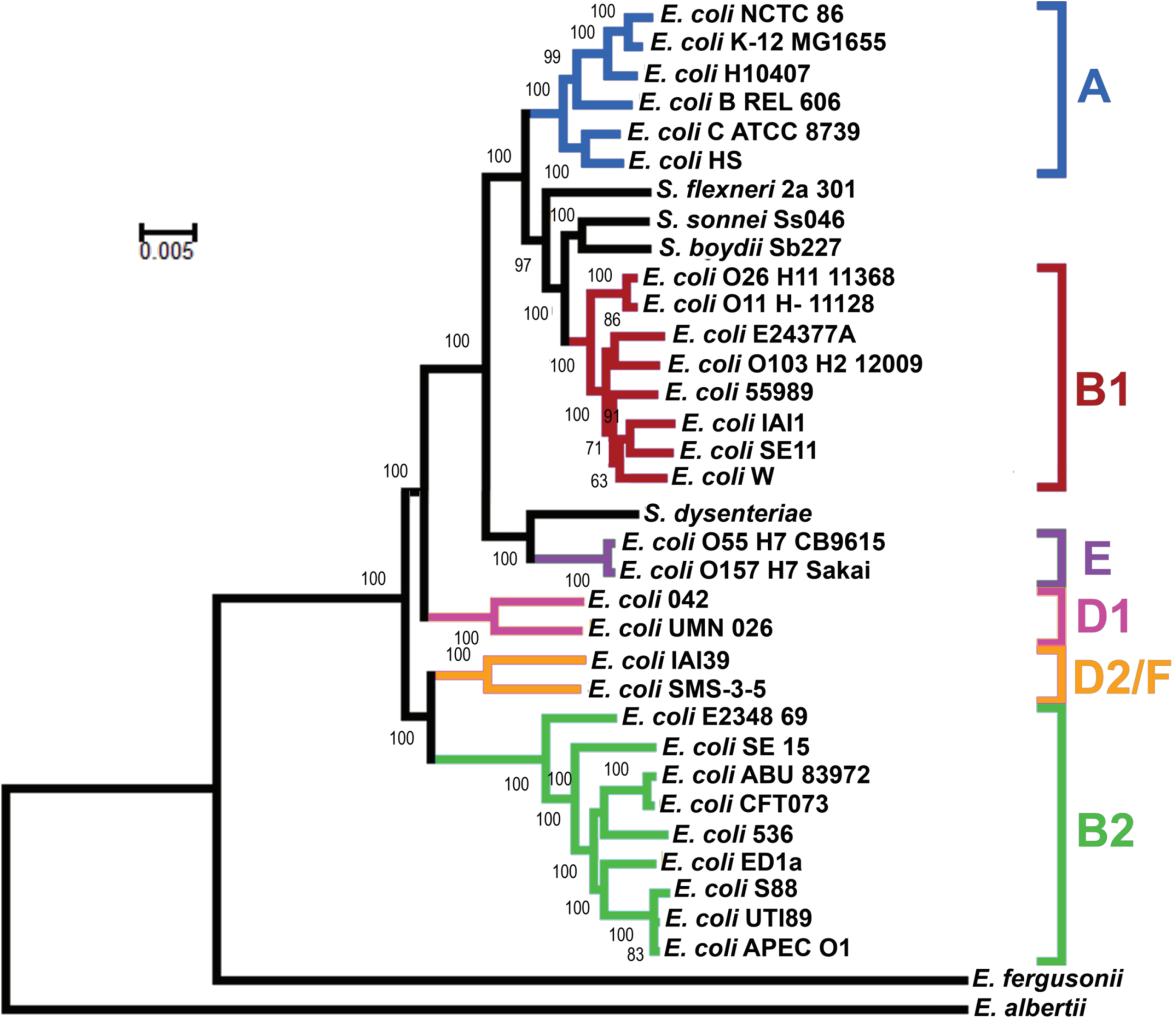
Phylogenetic relationships amongst sequenced *E. coli* genomes. The genomes of *E. albertii* and *E. fergusonii* are included as outgroup sequences. The tree was obtained by maximum likelihood analysis of a concatenated alignment of 2173 genes, using the general time reversible (GTR/REV) model, with the CAT approximation of rate heterogeneity as implemented in RAxML version 8.1.3. The numbers on individual branches indicate the percentage support from 100 non-parametric bootstrap replicates, performed using the rapid algorithm implemented in RAxML. T﻿he branch length indicates the number of substitutions per site. The major *E. coli* phylogenetic groups are indicated.

The phylogeny of *E. coli* NCTC86 was resolved through comparisons with the genome sequences of other *E. coli* and *Shigella* strains. The core genome was used to reconstruct a phylogenetic tree (Fig. 3), with *Escherichia albertii* and *Escherichia fergusonii* included as outgroups. As previously noted, the *E. coli* subgroups A, B1, B2, D and E are all monophyletic, with the exception of group D; group D is divided at the root [12]. *E. coli* NCTC 86 is located in the A subgroup and clusters closely with the non-pathogenic laboratory strains of *E. coli* K-12. *E. coli* K-12 is considered a commensal derivative on the basis that it was isolated from a patient with diphtheria and without diarrhoeal disease or urinary tract infections [13]. Thus, the location of *E. coli* NCTC 86 close to *E. coli* K-12 is consistent with Escherich’s observations of a non-pathogenic organism that was part of the normal microbiota [7].

A recurrent issue in the field of *E. coli* biology has been the assumption that *E. coli* K-12 represents an ancestral evolutionary lineage of the species. This misplaced belief probably arose from the fact *E. coli* K-12 was an early isolate of the species. Further confusion arises when the genomes of pathogenic *E. coli* are considered: *E. coli* K-12 is often used as a baseline genome with the simplistic view that pathogens are *E. coli* K-12 derivatives that have acquired extra DNA encoding disease-causing functions. These observations have been challenged before [14], and are again challenged here. Despite their early isolation, it is clear from their phylogenetic distribution that neither *E. coli* K-12 nor *E. coli* NCTC 86 represents an ancestral state for the species. Indeed, the observation that these strains are distant from the root, and the smaller size of their genetic complement, suggest these strains are undergoing reductive evolution as they transition to a state of commensalism. Similar reductive evolution has been noted for many intracellular obligate organisms as they shed their free-living lifestyles in favour of a less competitive existence in a host-restricted environment [15, 16]. Genetic attrition can also be observed for *E. coli* NCTC 86. For example, the locus encoding ETT2 (ROD 24), a cryptic type 3 secretion system (T3SS), appears to be intact in *E. coli* strains O157 : H7 and 042, but like other *E. coli* strains, the locus is severely eroded in *E. coli* NCTC 86 (Fig. S1). In a similar manner, the majority of the locus encoding Flag-2, a cryptic lateral flagellum found in *E. coli* 042 and UMN026, is absent in *E. coli* NCTC 86 (Fig. S2). Such observations support the hypothesis that *E. coli* NCTC 86 is a modern rather than an ancestral lineage of *E. coli*.

### Comparative genomics of *E. coli* NCTC 86

**Fig. 4.**
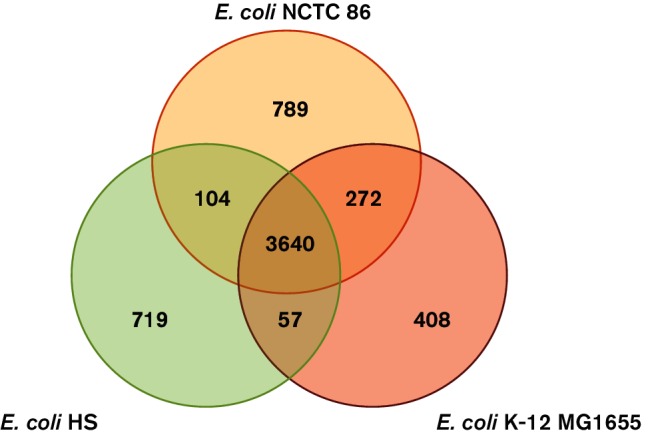
Comparison of the genetic content of *E. coli* NCTC 86 with other genome-sequenced *E. coli*. The three strains belong to the same phylogenetic group (group A) and share 3640 common genes. *E. coli* HS and NCTC 86 share 104 genes that are not present in *E. coli* K-12. Similarly, *E. coli* K-12 has 272 genes in common with *E. coli* NCTC 86 that are absent in *E. coli* HS. *E. coli* HS and K-12 share 57 genes that are absent from *E. coli* NCTC 86. All strains possess a complement of unique genes not present in the other strains; there are 789 genes present in *E. coli* NCTC 86 not present in *E. coli* K-12 or *E. coli* HS, 719 genes present in *E. coli* HS not present in *E. coli* K-12 and NCTC 86, and 408 genes in *E. coli* K-12 not found in *E coli* HS or NCTC 86.

As previously stated, phylogenetic mapping revealed *E. coli* NCTC 86 is located in the A subgroup along with the non-pathogenic laboratory strain *E. coli* K-12 and the commensal isolate *E. coli* HS. Comparison of *E. coli* NCTC 86 with the closely related *E. coli* K-12 and HS strains revealed that these chromosomes are largely similar, with 3640 genes conserved in all three strains (Fig. 4). Analyses of the gene contents of all three strains revealed that *E. coli* NCTC 86 contains 789 CDSs that are not present in the other two non-pathogenic strains. The majority (54 %) of these genes are predicted to encode transposons, integrases, prophage genes and other mobility factors or hypothetical proteins that are located throughout the chromosome. These 601 CDSs are contained in RODs distributed across the genome: they are discussed in the sections below. *E. coli* HS and NCTC 86 share 104 common genes not present in *E. coli* K-12, the majority of which encode hypothetical proteins. The rest contain a variety of genes, such as a type 6 secretion system (T6SS) NCTC86_02960–76. In contrast, there are 272 genes present in *E. coli* K-12 and NCTC 86 not found in *E. coli* HS. Around half of these are genes of unknown function; the rest are an assortment of genes such as the *fecABCDEIR* genes, which encode a ferric citrate uptake system, and the *wcaABCDEFGHIJKLMN* genes, which encode proteins involved in colanic acid synthesis. Additionally, *E. coli* HS and K-12 contain 57 genes not found in *E. coli* NCTC 86, over half of which consist of genes of unknown function or mobile elements. The rest are an assortment of genes such as the cold shock protein genes, *cspBSHI*, and the antibiotic-resistance genes, such as *acrR* and *blr* (Table S2). *E. coli* K-12 contains 408 genes not present in either *E. coli* NCTC 86 or HS. Again, most of these are mobile elements.

### Serotype antigens

Three major structures associated with the cell envelope of Gram-negative bacteria are flagella, lipopolysaccharide (LPS) and capsular polysaccharide (CPS), which mediate motility and protect the organism by providing a barrier to noxious substances and components of the immune system, respectively. The flagella, LPS and CPS are the three major antigenic determinants of *E. coli*: named the H-, O- and K-antigens, respectively. These structures can demonstrate high degrees of polymorphism that give rise to differences in antigenicity. This polymorphism has been exploited for many years for the serological detection and typing of *E. coli* isolates [17].

Flagellin (FliC) is the major protein subunit of the flagellum. Amino acid variation in flagellin is responsible for changes to the antigenic profile of the flagellum. Examination of the *E. coli* NCTC 86 genome sequence revealed the that major flagellar locus encodes an H10-serotype flagellin subunit that is identical to the flagellar locus of *E. coli* Bi 623–42, an O11 : H10 strain isolated from a patient with peritonitis [18]. This is consistent with the detection of the H10 flagellin reported for *E. coli* NCTC 86 [19].

Smooth LPS is a repeating structure termed the O-antigen side chain polysaccharide, which is chemically linked to the core oligosaccharide. The core oligosaccharide is further divided into the inner and outer core. In *E. coli*, the genes encoding the lipid A-core oligosaccharide and the O-antigen polysaccharide portions of LPS are encoded separately on the chromosome and are synthesized by independent pathways before linkage at the inner membrane [20]. The inner core and lipid A portions of the molecule are highly conserved. The outer core exhibits variation giving rise to five different core oligosaccharide structures designated K-12, R1, R2, R3 and R4. Inspection of the *E. coli* NCTC 86 genome revealed a locus (ROD 37) encoding an R2 oligosaccharide core. This locus is 99 % identical over 8 kb to the R2 prototype strain *E. coli* F632, which is an O-antigen-deficient derivative of *E. coli* 100 : K?(B) : H2 [21]. The biological significance of the R2 core is unknown: it is the least frequently occurring core type amongst commensal and pathogenic *E. coli* [22]. However, the presence of an R2 oligosaccharide core in a commensal organism suggests that efforts to target vaccines to this core region might be unwise, as it might negatively impact on the ability of a commensal organism to colonize the gut [23].

In striking contrast to the lipid A-core components, the O-antigens of LPS are chemically and structurally diverse. Over 180 serologically distinct O-antigens have been identified in *E. coli* [24]. Scrutiny of the *E. coli* NCTC 86 genome revealed a locus encoding the proteins to produce an O-antigen of serotype O15 (ROD 4). This locus is 99 % identical to the nucleotide sequence of the O-antigen locus from *E. coli* G1201 (O15 : K14 : H4) over 9.2 kb. Analysis of the O-antigen-encoding locus revealed that the *E. coli* NCTC 86 locus is 2.3 kb smaller than the *E. coli* G1201 locus. This deletion appears to have occurred through recombination of the ISE3C element with the *wzy* gene, which encodes the O-antigen polymerase, resulting in truncation of *wzy* and loss of the *wzx* and *wbuS* genes encoding the O-antigen flippase and a glycosyltransferase, respectively [25–27]. The loss of O-antigen expression in *E. coli* is a common feature of strains cultured in the laboratory for extended periods of time [28, 29].

Capsules are a series of high-molecular-mass polysaccharides that coat the bacterial surface. They are widely distributed and are found in many diverse pathogens such as *Neisseria meningitidis*, *Staphylococcus aureus, Streptococcus pneumoniae* and *E. coli*. At least 80 different polysaccharide capsules have been identified in *E. coli*. They have been separated into four different types (groups 1–4) based on their genetic organization, as well as their physical and biochemical properties [30]. *E. coli* NCTC 86 possesses the *kps* locus on ROD 31 (Fig. S3). The arrangement of the *kpsFEDUCS* capsular biosynthesis genes in region I and the ABC transporter genes *kpsMT* in region III are consistent with the production of group 2 CPS. The variable region II, which is the determinant of antigenicity, is 98 % identical to the genes in region II of *E. coli* CFT073 (Fig. S3). This suggests that the *E. coli* NCTC 86 *kps* locus encodes a K-2 serotype capsule. Such capsules are often associated with strains that cause extra-intestinal infection, where they provide protection against complement-mediated killing. However, as commensals do not need to evade the immune system, it might play a role in protection in the environment, such as desiccation.

### Metal binding and resistance

Iron plays an essential role in metabolic and cellular processes, in particular acting as a cofactor for several critical enzymes [31]. However, free iron is not readily available. In an aqueous environment at physiological pH it forms insoluble Fe(OH)_3_. Pathogens and commensals encounter additional problems *in vivo* as iron is extensively chelated by host proteins, such as ferritin and lactoferrin [32]. Thus, bacteria require efficient iron-acquisition mechanisms in order to survive. Some bacteria secrete molecules termed siderophores that have high affinity for iron. The iron-bound forms of the siderophore are recognized by specific proteinaceous machines that facilitate their uptake into the bacterial cell [33]. *E. coli* NCTC 86 possesses a locus, located on ROD 5, which encodes the siderophore yersiniabactin (Ybt) (Fig. S4). Ybt was first described in *Yersinia*, but it is widespread amongst the Enterobacteriaceae [34]. The *E. coli* NCTC 86 locus is most similar to the *E. coli* 042 locus (97 % identity over 24 kb). Interestingly, in addition to the reported iron-acquisition functions of Ybt, some studies suggest that Ybt contributes to bacterial resistance to copper [35]. Copper ions have been shown to be toxic to *E. coli* [36, 37]. Therefore, Ybt might act as a countermeasure to copper toxicity by sequestering host-derived copper and preventing its catechol-mediated reduction to copper (I), this enables bacteria to proliferate *in vivo* and can confer protection from redox-based phagocytic defences [38].

Like copper, silver ions show antibacterial activity against *E. coli*. Interestingly, *E. coli* NCTC 86 harbours a 36 kb locus (*sil*) comprised of nine ORFs (ROD36) that are associated with silver resistance (Fig. S5). The *sil* locus confers resistance though a sliver ion efflux system. Although the *sil* locus is frequently found on plasmids harbouring antibiotic-resistance genes, such as the pMG101 plasmid [39], in *E. coli* NCTC 86 it was found on the chromosome. The locus had a similar genetic arrangement to *E. coli* C ATCC8739, H10407 and in *E. coli* 55989, *Enterobacter cloacae* ATCC 13047 and *Cronobacter sakazakii* CMCC 45402 (Fig. S5), where it is also found on the chromosome. It was suggested that this locus was only recently acquired by *E. coli* after the introduction of silver nanoparticles as an antibacterial agent. This hypothesis was based on the observation that *E. coli* strains recently isolated from humans possessed the *sil* locus, but that no *E. coli* strain of an avian origin was found to contain the locus [40]. However, *E. coli* NCTC 86 clearly acquired this locus before the current widespread use of silver nanoparticles, suggesting there is an alternative explanation for the appearance of this locus in human isolates. It is tempting to speculate that the reason *E. coli* NCTC 86 possesses the *sil* locus was the widespread use of silver utensils and currency in Germany in the 1800s.

### Metabolism

The metabolic properties encoded in a bacterium are one of the primary factors in determining whether it is successful in an ecological niche. *E. coli* NCTC 86 contains a variety of RODs encoding loci associated with metabolic functions, such as ethanolamine utilization, a set of acyl-CoA synthetase and permease genes, and putative C_4_-dicarboxylate and sugar-uptake systems.

The phosphotransferase system (PTS) is a translocation system that transports a variety of different sugars into the cell. The PTS consists of two proteins, an enzyme I and HPr, and a number of carbohydrate-specific enzymes, the enzymes II. In addition to the PTSs encoded in the conserved core *E. coli* genes, *E. coli* NCTC 86 possesses two sugar-uptake systems encoded on RODs. The *vpe* locus is located on ROD 40 and is 99.77 % identical to the *vpe* locus of *E. coli* O104 : H4 2009EL-2050 strain. The *vpe* operon was first described in the uropathogenic *E. coli* (UPEC) strain AL511 in which mutants were shown to produce much smaller amounts of group two capsule [41]. As noted earlier, *E. coli* NCTC 86 encodes a similar group two capsule. ROD 40 also contains the *deoK* operon. This region conferring the ability to utilize deoxyribose as a carbon source is thought to have been transferred from *Salmonella enterica* to *E. coli* [42]. The *deoK* operon is commonly connected with strains isolated from infected blood and urine, in which it is usually found in extensive islands carrying genes contributing to the adaptive properties and/or virulence of UPEC strains, such as the sepsis strain *E. coli* AL862 [43]. The second putative PTS is located on ROD 34 of *E. coli* NCTC 86 and is 98 % identical to that in *E. coli* O157 : H7 Sakai. However, the function of this system is unknown.

*E. coli* NCTC 86 contains a putative C_4_-dicarboxylate uptake (Duc) transporter system located on ROD 30 that is composed of three genes clustered together: a tripartite ATP-independent periplasmic (TRAP) transporter; an ABC transporter permease; and a substrate-binding protein. *E. coli* NCTC 86 system is 100 % identical to the C_4_-dicarboxylate system found in *E. coli* IAI39. However, in *E. coli* IAI39 there is a transposon inserted in the centre of the periplasmic (TRAP) transporter gene, indicating that it might not be functional in this isolate. While many bacteria utilize molecules such as aspartate, malate, fumarate and succinate for anaerobic respiration (aspartate is metabolized under both aerobic and anaerobic conditions), the function and substrate of this specific C_4_-dicarboxylate uptake system remains unknown.

### Protein secretion systems

Protein secretion involves translocation across the cytoplasmic membrane, and in Gram-negative bacteria the additional barriers of a peptidoglycan-filled periplasm and an outer membrane. To combat these latter obstacles, Gram-negative bacteria have evolved a number of highly specialized protein secretion systems [44]. Inspection of the *E. coli* NCTC 86 genome revealed loci encoding a type 1 secretion system (T1SS), type 2 secretion system (T2SS), T3SS, type 5 secretion system (T5SS) and a T6SS. The ETT2 and Flag-2 T3SSs were discussed earlier.

Conventional T1SSs of Gram-negative bacteria are composed of a secreted substrate protein and three envelope-associated subunits: a TolC-like outer membrane pore-forming protein (OMP), and two inner-membrane-associated proteins, termed the ATP-binding cassette (ABC) protein, which provide energy to the system, and the membrane-fusion protein (MFP), which contacts with the TolC-like protein to form the secretion channel across the cell envelope on ROD 17 [45]. Like the conventional systems, the *E. coli* NCTC 86 system possesses an OMP (NCTC86_02172), MFP (NCTC86_02173) and ABC protein (NCTC86_02170). In contrast to the conventional T1SSs, the locus also encodes an additional ABC protein (NCTC86_02173) and an additional component whose function is unknown (NCTC86_02170) (Fig. S6). Unlike the conventional T1SS, in which the C-terminal sequence is uncleaved, the putative substrate molecules for the unconventional systems possess an N-terminal Sec-dependent signal sequence that is cleaved. Located downstream of the genes encoding the translocation machinery is a pseudogene that would have encoded a substrate molecule (NCTC86_02180) but is disrupted by the insertion of genes encoding transposases. However, located 11.2 kb upstream is NCTC86_02122, which appears to encode a complete putative substrate molecule. This secretion system is homologous to the Aat/dispersin previously described for enteroaggregative *E. coli* 042. In *E. coli* 042, the substrate molecule is localized to the bacterial cell surface where it promotes fimbrial-mediated adherence by altering the surface charge of the bacterium [46]. The presence of this gene cluster in a commensal bacterium suggests its primary role is in colonization rather than in pathogenesis as previously suggested [47].

Several pathogenic strains of *E. coli* possess a chromosomally encoded T2SS. For enterotoxigenic *E. coli* it is essential for secretion of heat-labile enterotoxin (LT) [48]. In *E. coli* K-12, the locus has undergone genetic attrition. It contains *yghJ–gspO* (*pppA*) – *gspC* (*yghF*) and the distal *gspL–gspM* genes, but not the remainder of the T2SS (Fig S7). *E. coli* NCTC 86 also possesses the distal *gspL–gspM* genes. However, the T2SS locus appears to be in an even later stage of genetic attrition. The *yghJGF* and *pppA* genes have also been lost. The erosion of this locus is further evidence for the adaptation of *E. coli* NCTC 86 to a commensal lifestyle.

T5SSs are a large and diverse superfamily of proteins. Based on structural differences and variations in the mode of biogenesis, T5SS has been divided into five subclasses: classical autotransporters (T5aSS); the two-partner secretion systems (T5bSS); the trimeric autotransporters (T5cSS); the chimeric autotransporters (T5dSS); and the inverted autotransporters (T5eSS). *E. coli* NCTC 86 does not appear to possess members of the T5bSS, T5cSS or T5dSS subfamilies. However, *E. coli* NCTC 86 possesses 15 loci encoding polypeptides with homology to the T5aSS subfamily and 3 polypeptides with homology to the T5eSS subfamily (Fig S8). Of the 15 T5aSS-encoding loci, 9 are found in *E. coli* K-12 and are scattered throughout the chromosome; 4 of these are pseudogenes. Remarkably, *E. coli* NCTC 86 possesses five loci (NCTC86_00823, NCTC86_02105, NCTC86_02886, NCTC86_03415 and NCTC86_04998) encoding polypeptides with homology to the autotransporter antigen 43. These loci are found on RODs 5, 17, 21, 31 and 41, although one (ROD 31; NCTC86_03415) is a pseudogene. These classical autotransporters have been implicated in adherence and biofilm formation. The three loci encoding members of the T5eSS subfamily can also be found in *E. coli* K-12. This family of proteins has been implicated in mediating attachment to host cells [49]. Scrutiny of the distribution of the T5SS genes across the evolutionary spectrum of *E. coli* reveals no strong association of these genes with pathogenic or non-pathogenic isolates. This suggests that they do not play a specific role in pathogenesis but rather promote colonization, a function that is consistent with their presence in a commensal strain.

*E. coli* NCTC 86 contains a T6SS. This locus encodes the Hcp and Vgr proteins that form a needle-like injection device and are essential for the T6SS to function. The locus also contains the TssA, E, F, G and K core component proteins. It is this type of T6SS that is most widespread amongst *E. coli* [50–52]. This 30 kb locus is located on ROD 22 and is >97 % identical to the T6SS of *E. coli* 55989, and is highly homologous to loci in *E. coli* 042, urinary tract isolates UMN026, 536 and UTI89, and avian isolates APEC O1 (Fig. S9). T6SSs have been shown to be widespread among the proteobacteria and have roles in inhibiting eukaryote cell division as well as possessing antibacterial properties, by acting as a mechanism to attack other bacterial cells [53]. Therefore, it is likely the locus provides *E. coli* NCTC 86 with a colonization advantage within the host.

**Fig. 5.**
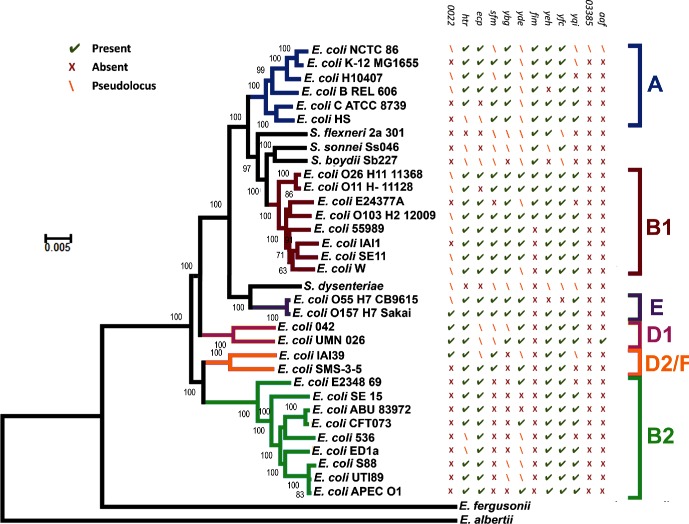
Genetic architecture and distribution of the CU systems of *E. coli* NCTC 86. Phylogenetic distribution of the fimbrial loci amongst pathogenic and non-pathogenic lineages of *E. coli*. The loci encoding the fimbrial systems demonstrate a differential distribution amongst the *E. coli* phylogeny. The branch lengths indicate the number of substitutions per site.

Proteobacteria use chaperone–usher (CU) pathways to assemble fimbriae on the bacterial surface. The CU system is divided into six subfamilies, designated α-, β-, γ-, κ-, π- and σ-fimbriae [54]. *E. coli* NCTC 86 contains 12 CU systems, 9 of which are also present in *E. coli* K-12 (Fig. 5). It lacks members of the κ-, β- or σ- subfamilies. As in *E. coli* K-12, these CU operons appear to be scattered throughout the chromosome. The other three CU systems in *E. coli* NCTC 86 (NCTC86_00020–6, NCTC86_03382–8 and NCTC86_02125–9) are found on RODs 1, 17 and 31, respectively. The CU system encoded on ROD 1 is homologous to the *mat* fimbriae of *E. coli* O157, but the first gene in this locus is truncated in *E. coli* NCTC 86 rendering the system non-functional. The CU locus on ROD 31 is disrupted by the insertion of two transposable elements and is unlikely to be functional. The locus on ROD 17 also encodes a non-functional CU system with homology to the aggregative adherence fimbriae of *E. coli* 042. There is no obvious phylogenetic signature for the presence or absence of the CU systems. Furthermore, there is no obvious association with pathogenic or non-pathogenic lineages, with the exception of shigellae, which appear to have lost many of the loci encoding these CU systems. As with the T5SS, these data suggest that the CU systems do not play a specific role in pathogenesis, but rather promote colonization. The diversity of systems may allow colonization of different hosts or colonization of different niches within the same host.

### Conclusions

The complete genetic content of *E. coli* NCTC 86 described in this article provides a modern interpretation for observations made by Escherich over 125 years ago. During the preparation of this manuscript, a draft sequence of the same strain and its historical provenance were reported [55]. These studies demonstrate that *E. coli* NCTC 86 is phylogenetically closely related to other commensal *E. coli* strains. The anthropocentric view of bacteriology has largely driven the study of pathogenic *E. coli* at the expense of understanding commensalism and, perhaps, of truly understanding the repertoire of genes that are required for pathogenesis and those which are simply required for colonization. The current study adds to the body of literature underpinning the study of commensal *E. coli* and hints that these organisms arose by reductive evolution as they adapted to an intimate life with their mammalian hosts. However, given the plasticity of the *E. coli* genome, further genomic studies are essential to determine those factors that are widely conserved for commensal *E. coli* from geographically diverse locations and distinctive human populations. With such studies, *E. coli* will undoubtedly remain as a model organism.

## Data bibliography

Aslett MA. GenBank accession number FN554766 (2009).
Aslett MA. GenBank accession number FN554767 (2009).
Brzuszkiewicz E, Brueggemann H, Liesegang H, Emmerth M, Oelschlaeger T *et al.* GenBank accession number CP000247 (2006).
Genoscope CEA. GenBank accession number CU928145 (2008).
Brzuszkiewicz EB, Liesegang H, Zdziarski J, Svanborg C, Hacker J *et al*. GenBank accession number CP001671 (2009).
Brzuszkiewicz EB, Liesegang H, Zdziarski J, Svanborg C, Hacker J *et al*. GenBank accession number CP001833 (2009).
Johnson TJ, Nolan LK. GenBank accession number CP000468 (2009).
Johnson TJ, Nolan LK. GenBank accession number DQ381420 (2006).
Johnson TJ, Nolan LK. GenBank accession number DQ517526 (2006).
Kim JF, Jeong H, Choi S-H, Yu D-S, Hur C-G *et al*. GenBank accession number CP000819 (2007).
Copeland A, Lucas S, Lapidus A, Glavina del Rio T, Dalin E *et al.* GenBank accession number CP000946 (2008).
Welch RA, Burland V, Plunkett GD III, Redford P, Roesch P *et al*. GenBank accession number AE014075 (2002).
Thomson NR. GenBank accession number FM180568 (2008).
Thomson NR. GenBank accession number FM180569 (2008).
Thomson NR. GenBank accession number FM180570 (2008).
Rasko DA, Rosovitz MJ, Brinkley C, Myers GSA, Seshadri R *et al*. GenBank accession number CP000795 (2007).
Rasko DA, Rosovitz MJ, Brinkley C, Myers GSA, Seshadri R *et al*. GenBank accession number CP000796 (2007).
Rasko DA, Rosovitz MJ, Brinkley C, Myers GSA, Seshadri R *et al*. GenBank accession number CP000797 (2007).
Rasko DA, Rosovitz MJ, Brinkley C, Myers GSA, Seshadri R *et al*. GenBank accession number CP000798 (2007).
Rasko DA, Rosovitz MJ, Brinkley C, Myers GSA, Seshadri R *et al*. GenBank accession number CP000799 (2007).
Rasko DA, Rosovitz MJ, Brinkley C, Myers GSA, Seshadri R *et al*. GenBank accession number CP000800 (2007).
Rasko DA, Rosovitz MJ, Brinkley C, Myers GSA, Seshadri R *et al*. GenBank accession number CP000801 (2007).
Fiedoruk K, Daniluk T, Swiecicka I, Murawska E, Sciepuk M *et al*. GenBank accession number CP007025 (2013).
Genoscope CEA. GenBank accession number CU928162 (2008).
Genoscope CEA. GenBank accession number CU928144 (2008).
Genoscope CEA. GenBank accession number CU928158 (2008).
Archer CT, Kim JF, Jeong H, Park J, Vickers CE *et al*. GenBank accession number CP002185 (2010).
Archer CT, Kim JF, Jeong H, Park J, Vickers CE *et al*. GenBank accession number CP002186 (2010).
Archer CT, Kim JF, Jeong H, Park J, Vickers CE, Lee S *et al*. GenBank accession number CP002187 (2010).
Aslett MA. GenBank accession number FN649414 (2009).
Aslett MA. GenBank accession number FN649415 (2009).
Aslett MA. GenBank accession number FN649416 (2009).
Aslett MA. GenBank accession number FN649417 (2009).
Aslett,M.A. GenBank accession number FN649418 (2009).
Rasko DA, Rosovitz M, Kaper JB, Nataro JP, Myers GS *et al*. GenBank accession number CP000802 (2007).
Genoscope CEA. GenBank accession number CU928160 (2008).
Genoscope CEA. GenBank accession number CU928164 (2008).
Blattner FR, Plunkett G III. GenBank accession number U00096 (2013).
Hattori M, Toh H, Oshima K, Yamashita A, Hayashi T *et al*. GenBank accession number AP010958 (2008).
Hattori M, Toh H, Oshima K, Yamashita A, Hayashi T *et al*. GenBank accession number AP010959 (2008).
Hattori M, Toh H, Oshima K, Yamashita A, Hayashi T *et al*. GenBank accession number AP010960 (2008).
Hattori M, Toh H, Oshima K, Yamashita A, Hayashi T *et al*. GenBank accession number AP010961 (2008).
Hattori M, Toh H, Oshima K, Yamashita A, Hayashi T *et al*. GenBank accession number AP010962 (2008).
Hattori M, Toh H, Oshima K, Yamashita A, Hayashi T *et al*. GenBank accession number AP010963 (2008).
Hattori M, Toh H, Oshima K, Yamashita A, Hayashi T *et al.* GenBank accession number AP010964 (2008).
Hattori M, Toh H, Oshima K, Yamashita A, Hayashi T *et al*. GenBank accession number AP010965 (2008).
Makino K. GenBank accession number AB011548 (1998).
Makino K. GenBank accession number AB011549 (1998).
Hattori M, Ishii K, Shiba T. GenBank accession number BA000007 (2000).
Hattori M, Toh H, Oshima K, Yamashita A, Hayashi T *et al*. GenBank accession number AP010953 (2008).
Hattori M, Toh H, Oshima K, Yamashita A, Hayashi T *et al*. GenBank accession number AP010954 (2008).
Hattori M, Toh H, Oshima K, Yamashita A, Hayashi T *et al*. GenBank accession number AP010955 (2008).
Hattori M, Toh H, Oshima K, Yamashita A, Hayashi T *et al*. GenBank accession number AP010956 (2008).
Hattori M, Toh H, Oshima K, Yamashita A, Hayashi T *et al*. GenBank accession number AP010957 (2008).
Wang L, Zhou Z, Li X, Liu B, Beutin L *et al*. GenBank accession number CP001846 (2009).
Wang L, Zhou Z, Li X, Liu B, Beutin L *et al*. GenBank accession number CP001847 (2009).
Genoscope CEA. GenBank accession number CU928146 (2008).
Genoscope CEA. GenBank accession number CU928161 (2008).
Jin Q, Yang F, Yang J, Jiang Y, Yan Y *et al*. GenBank accession number CP000036 (2004).
Yang J, Chen L, Jin Q. GenBank accession number CP000037 (2007).
Jin Q, Yang F, Yang J, Jiang Y, Yan Y *et al*. GenBank accession number CP000034 (2004).
Yang J, Chen L, Jin Q. GenBank accession number CP000035 (2007).
Yang J, Yang F, Chen L, Jin Q. GenBank accession number CP000640 (2007).
Hattori M, Oshima K, Toh H, Sasamoto H, Shiba T. GenBank accession number AP009240 (2006).
Hattori M, Oshima K, Toh H, Sasamoto H, Shiba T. GenBank accession number AP009241 (2006).
Hattori M, Oshima K, Toh H, Sasamoto H, Shiba T. GenBank accession number AP009242 (2006).
Hattori M, Oshima K, Toh H, Sasamoto H, Shiba T. GenBank accession number AP009243 (2006).
Hattori M, Oshima K, Toh H, Sasamoto H, Shiba T. GenBank accession number AP009244 (2006).
Hattori M, Oshima K, Toh H, Sasamoto H, Shiba T. GenBank accession number AP009245 (2006).
Hattori M, Oshima K, Toh H, Sasamoto H, Shiba T. GenBank accession number AP009246 (2006).
Hattori M, Yamashita A, Toh H, Oshima K, Shiba T. GenBank accession number AP009378 (2007).
Hattori M, Yamashita A, Toh H, Oshima K, Shiba T. GenBank accession number AP009379 (2007).
Jin Q, Shen Y, Wang JH, Liu H, Yang J *et al*. GenBank accession number AE005674 (2011).
74. Jin Q, Zhang JY, Liu H, Yang J, Yang F *et al*. GenBank accession number AF386526 (2007).
Fricke WF, Wright MS, Lindell AH, Harkins DM, Baker-Austin C *et al*. GenBank accession number CP000970 (2008).
Fricke WF, Wright MS, Lindell AH, Harkins DM, Baker-Austin C *et al*. GenBank accession number CP000971 (2008).
Fricke WF, Wright MS, Lindell AH, Harkins DM, Baker-Austin C *et al*. GenBank accession number CP000972 (2008).
Fricke WF, Wright MS, Lindell AH, Harkins DM, Baker-Austin C *et al*. GenBank accession number CP000973 (2008).
Fricke WF, Wright MS, Lindell AH, Harkins DM, Baker-Austin C *et al*. GenBank accession number CP000974 (2008).
Jin Q, Yang F, Yang J, Jiang Y, Yan Y *et al.* GenBank accession number CP000038 (2004).
Yang J, Chen L, Jin Q. GenBank accession number CP000039 (2007).
Yang J, Yang F, Chen L, Jin Q. GenBank accession number CP000641 (2007).
Yang J, Yang F, Chen L, Jin Q. GenBank accession number CP000642 (2007).
Yang J, Yang F, Chen L, Jin Q. GenBank accession number CP000643 (2007).
Genoscope CEA. GenBank accession number CU928148 (2008).
Genoscope CEA. GenBank accession number CU928149 (2008).
Genoscope CEA. GenBank accession number CU928163 (2008).
Chen SL, Hung C-S, Xu J, Reigstad CS, Magrini V *et al*. GenBank accession number CP000243 (2006).
Chen SL, Hung C-S, Xu J, Reigstad CS, Magrini V *et al*. GenBank accession number CP000244 (2006).

## Supplementary Data

2642 Supplementary File 1

## Supplementary Data

12 Supplementary File 2

## Supplementary Data

4355 Supplementary File 3
