# Supplementary material for: Sequencing a piece of history: complete genome sequence of the original Escherichia coli strain
Source: Microb Genom. 2017 Mar 23;3(3):mgen000106. doi: 10.1099/mgen.0.000106 (PMC5382810; doi:10.1099/mgen.0.000106)
Supplement: Supplementary File 1 [file mgen-3-106-s001.pdf]

Supplemental Figure 1: Alignment of the *E. coli* Type Three secretion system. Alignments reveal the genetic attrition of *E. coli* Type Three secretion system 2 (ETT2). ETT2 is located on ROD 24 in *E. coli* NCTC 86. ETT2 is most complete in *E. coli* O157:H7 and *E. coli* 042 but is in various stages of degradation in *E. coli* NCTC 86 and *E. coli* K-12. EET2 is completely absent in CFT073. The CDSs are indicated by arrows coloured according to the % GC content of the gene.

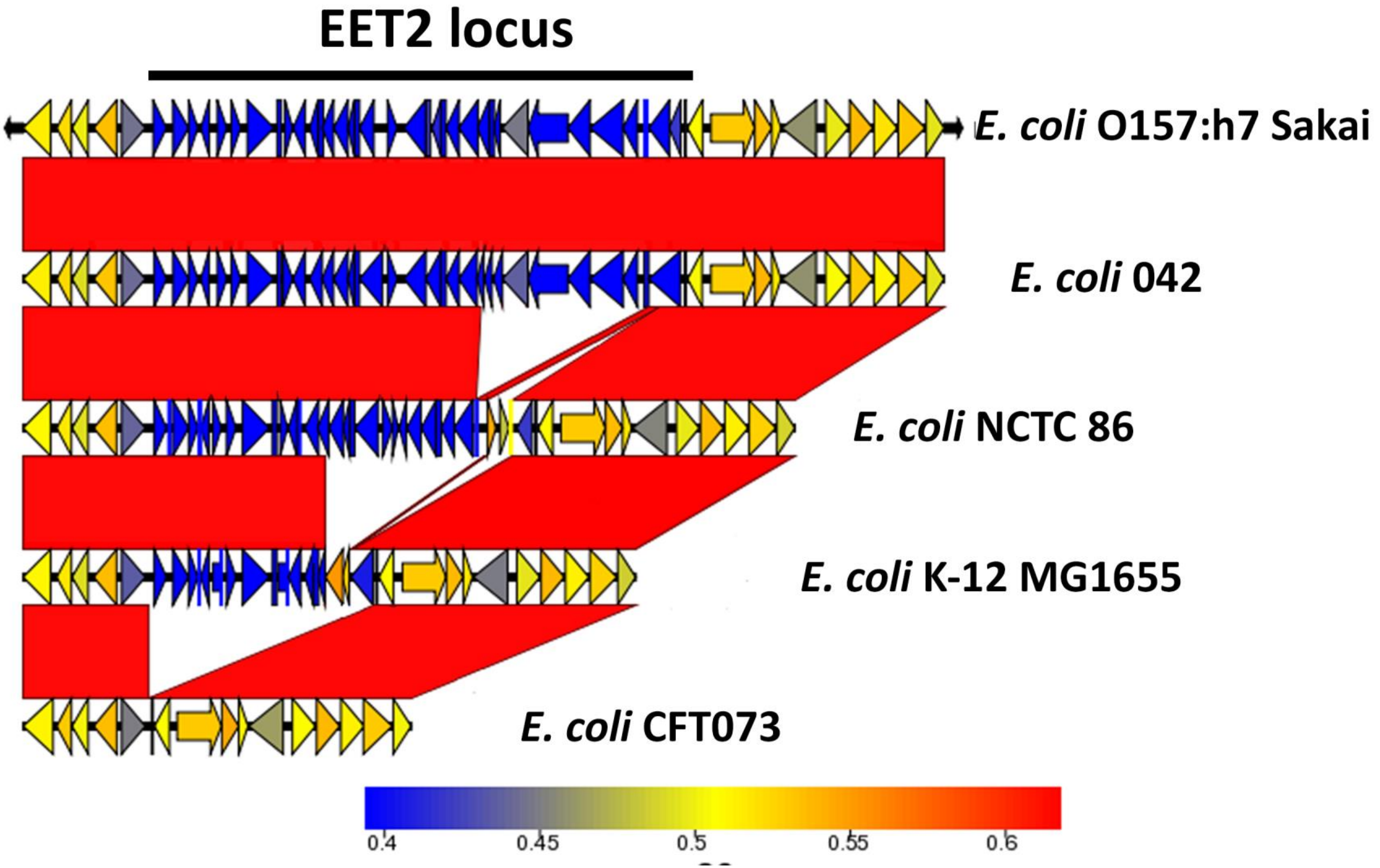
EET2 locus
E. coli O157:h7 Sakai
E. coli 042
E. coli NCTC 86
E. coli K-12 MG1655
E. coli CFT073
0.4
0.45
0.5
0.55
0.6

Supplemental Figure 2: Schematic representation of the Flag-2 gene cluster in *E. coli* 042. Schematic representation of the 48.8-kb region of the *E. coli* 042 genome encoding Flag-2. The illustration shows the absence of the locus when in *E. coli* NCTC 86. CDSs are indicated by arrows coloured according to the GC content of the gene.

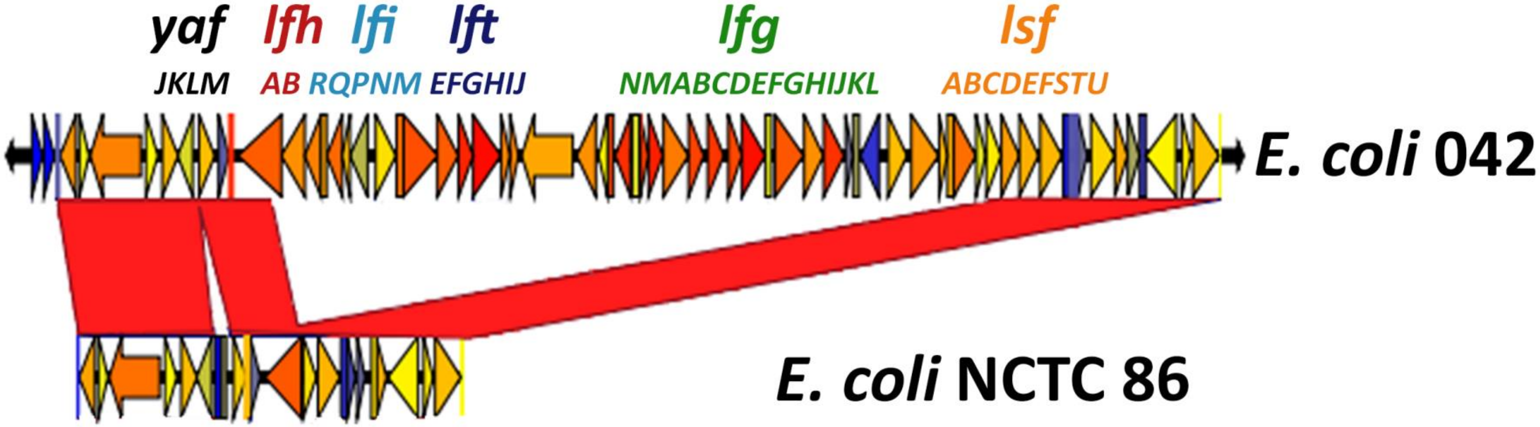
yaf lfh lfi lft lfg lsf
JKLM AB RQPNM EFGHIJ NMABCDEFGHIJKL ABCDEFSTU
E. coli 042
E. coli NCTC 86

Supplemental Figure 3: Alignment of the capsular operon from of *E. coli* CFT073 with *E. coli* NCTC 86. Alignments reveal genetic lesions in the *E. coli* NCTC 86 contains the same *kps* locus found on ROD 31, which encodes the capsule found in *E. coli* CTF073. The locus is divided into three regions. The conserved regions 1 and the variable Region 2 that encode enzymes for polymer biosynthesis, and the complexity of this region corresponds UPEC *E. coli* CFT073 group two capsule indicating it possesses the K-2 antigen. CDSs are indicated by arrows and their colour represents the GG content according to the scale below.

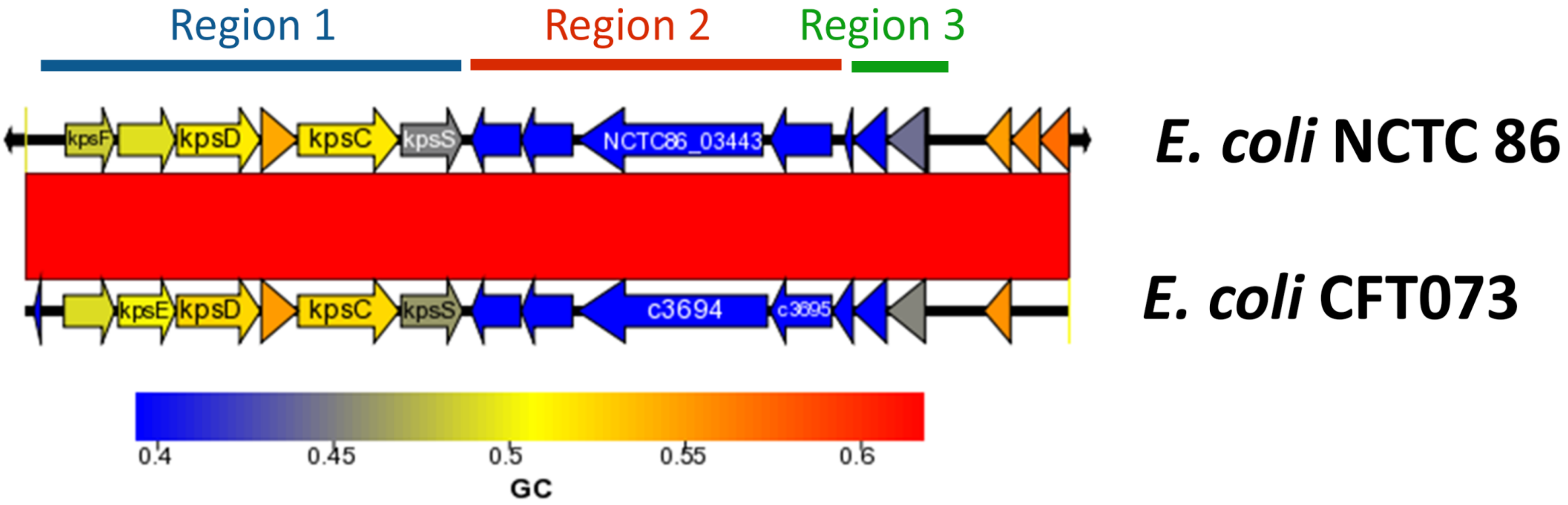
Region 1
Region 2
Region 3
kpsF
kpsD
kpsC
kpsS
NCTC86_03443
E. coli NCTC 86
kpsE
kpsD
kpsC
kpsS
c3694
c3695
E. coli CFT073
0.4
0.45
0.5
0.55
0.6
GC

Supplemental Figure 4: Comparison of the Yersiniabactin locus. Alignment of the Yersiniabatcin operon from of *Yersinia pestis* with *E. coli* NCTC 86, *E. coli APEC O1, E. coli* EAEC 042 *E. coli* K-12. Alignments reveal the locus in present in all strains apart from in the *E. coli* K12. The yersiniabactin locus is located on ROD 5 in *E. coli* NCTC 86. CDSs are indicated by arrows coloured according to the GC content of the gene revealing a high GC content.

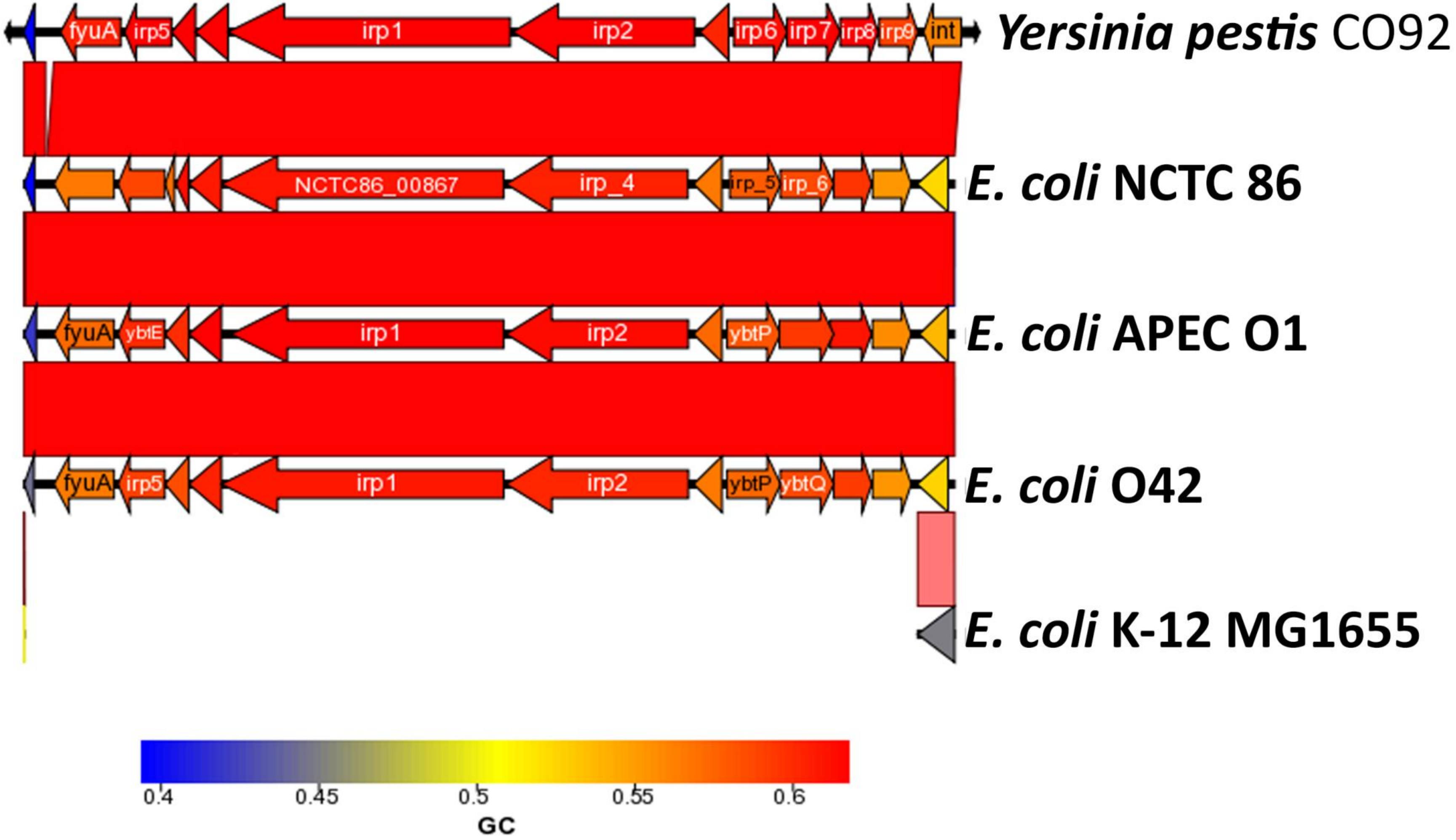
fyuA
irp5
irp1
irp2
irp6
irp7
irp8
irp9
int
Yersinia pestis CO92
NCTC86_00867
irp_4
irp_5
irp_6
E. coli NCTC 86
fyuA
ybtE
irp1
irp2
ybtP
E. coli APEC O1
fyuA
irp5
irp1
irp2
ybtP
ybtQ
E. coli O42
E. coli K-12 MG1655
0.4
0.45
0.5
0.55
0.6
GC

Supplemental Figure 5: Alignment of the *sil* locus confers resistance to silver. *E. coli* NCTC 86 *sil* locus that confers resistance to silver. Alignment of the *sil* locus shows that it is present in *E.* coli 55989, C str. ATTC 8739, H10407 as well as *E. coli* NCTC 86 where it is located on ROD 36. The *sil* operon is widespread and not confided to *E. coli,* it is also present in *Cronobacter sakazakil and Enterobacter coloace.* CDSs are indicated by arrows coloured according to the GC content of the gene.

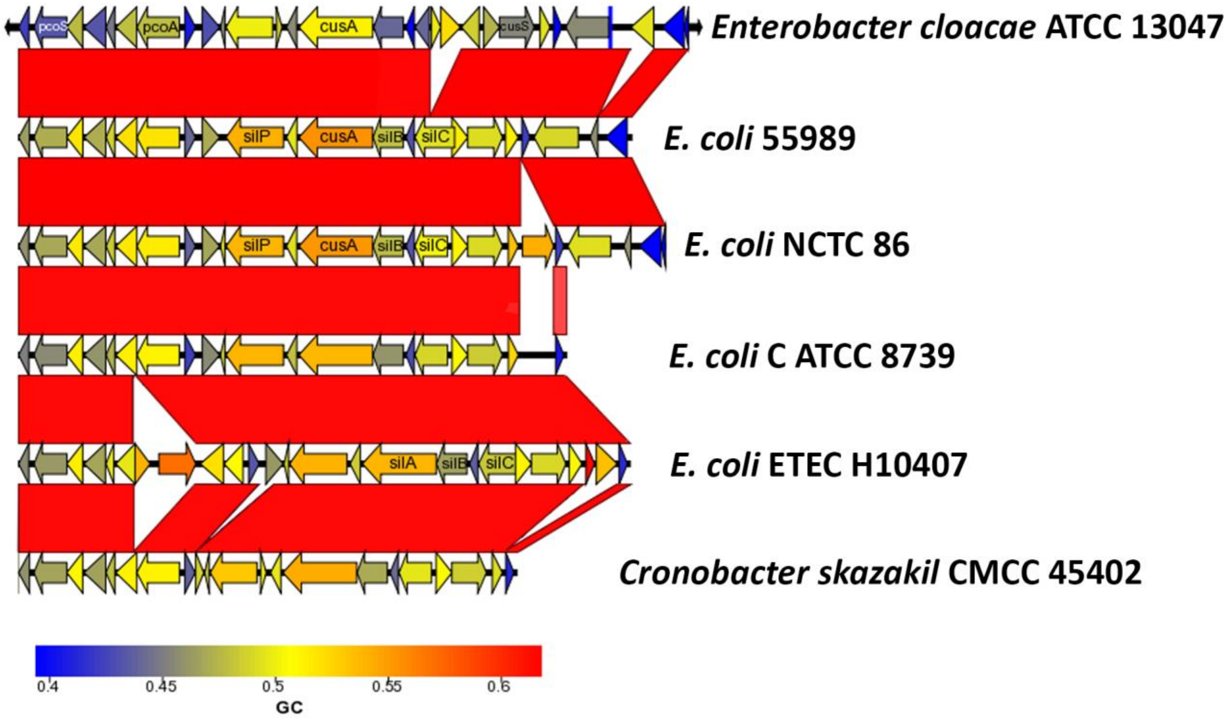

pcoS
pcoA
cusA
cusS
Enterobacter cloacae ATCC 13047
silP
cusA
silB
silC
E. coli 55989
silP
cusA
silB
silC
E. coli NCTC 86
E. coli C ATCC 8739
silA
silB
silC
E. coli ETEC H10407
Cronobacter skazakil CMCC 45402
0.4
0.45
0.5
0.55
0.6
GC

Supplemental Figure 6: Genomic architecture of the Type 1 Secretion System. Alignment of the *E. coli* NCTC 86 Type 1 secretion system to the EAEC 042 *aat* gene cluster. The loci share 63 - 81 % amino acid sequence identity and appear to differ in the efflux transporter/ *aatB* gene. CDSs are indicated by arrows coloured according to the GC content of the gene revealing a low GC content.

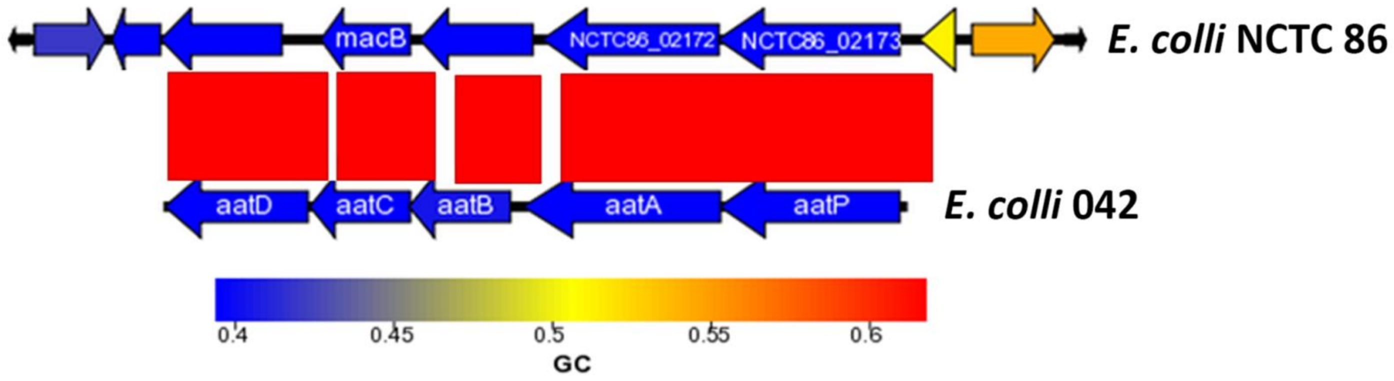
macB
NCTC86_02172
NCTC86_02173
E. colli NCTC 86
aatD
aatC
aatB
aatA
aatP
E. colli 042
0.4
0.45
0.5
0.55
0.6
GC

Supplemental Figure 7: Alignment Type 2 Secretion System *E. coli* NCTC 86 with *E. coli* 042, CFT073 an K-12. The *E. coli* NCTC 86 the type 2 secretion system is in a later stage of degradation in compared to *E. coli* K-12 however it does appear to still possess a set of acyl-CoA synthetase and permease genes absent from *E. coli* K-12 and appears to be in an identical state as the *E. coli* CFT073 genome. CDSs are indicated by arrows coloured according to the GC content of the gene.

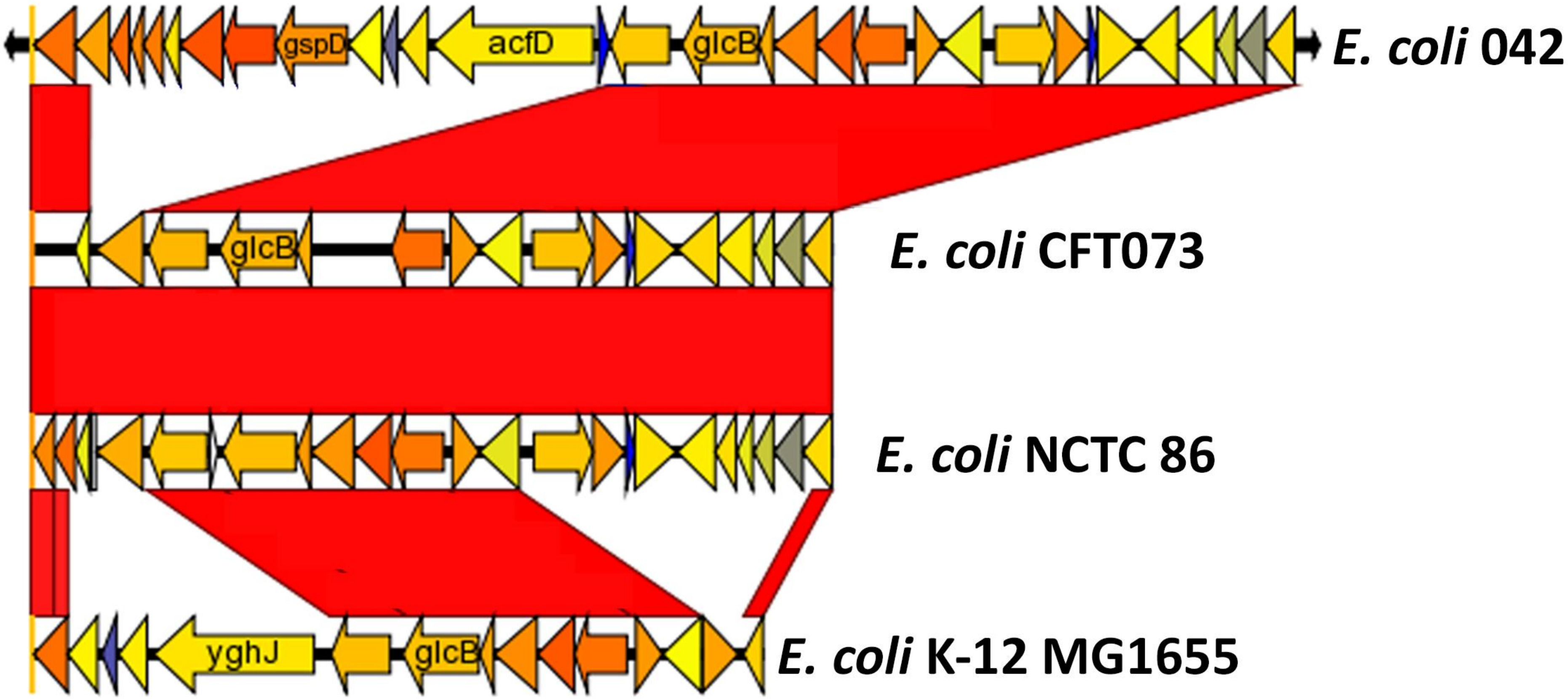
gspD
acfD
glcB
E. coli 042
glcB
E. coli CFT073
E. coli NCTC 86
yghJ
glcB
E. coli K-12 MG1655

Supplemental Figure 8: Phylogenetic distribution of members of the Type 5 secretion system present in NCTC 86. The Autotransporters are differentially represented amongst the *E. coli* phylogeny, though several members of this group show phylogenetic clustering. Their presence in both commensal and pathogenic lineages suggesting autotranspoters are not directly responsible for the ability of *E. coli* to mediate disease.

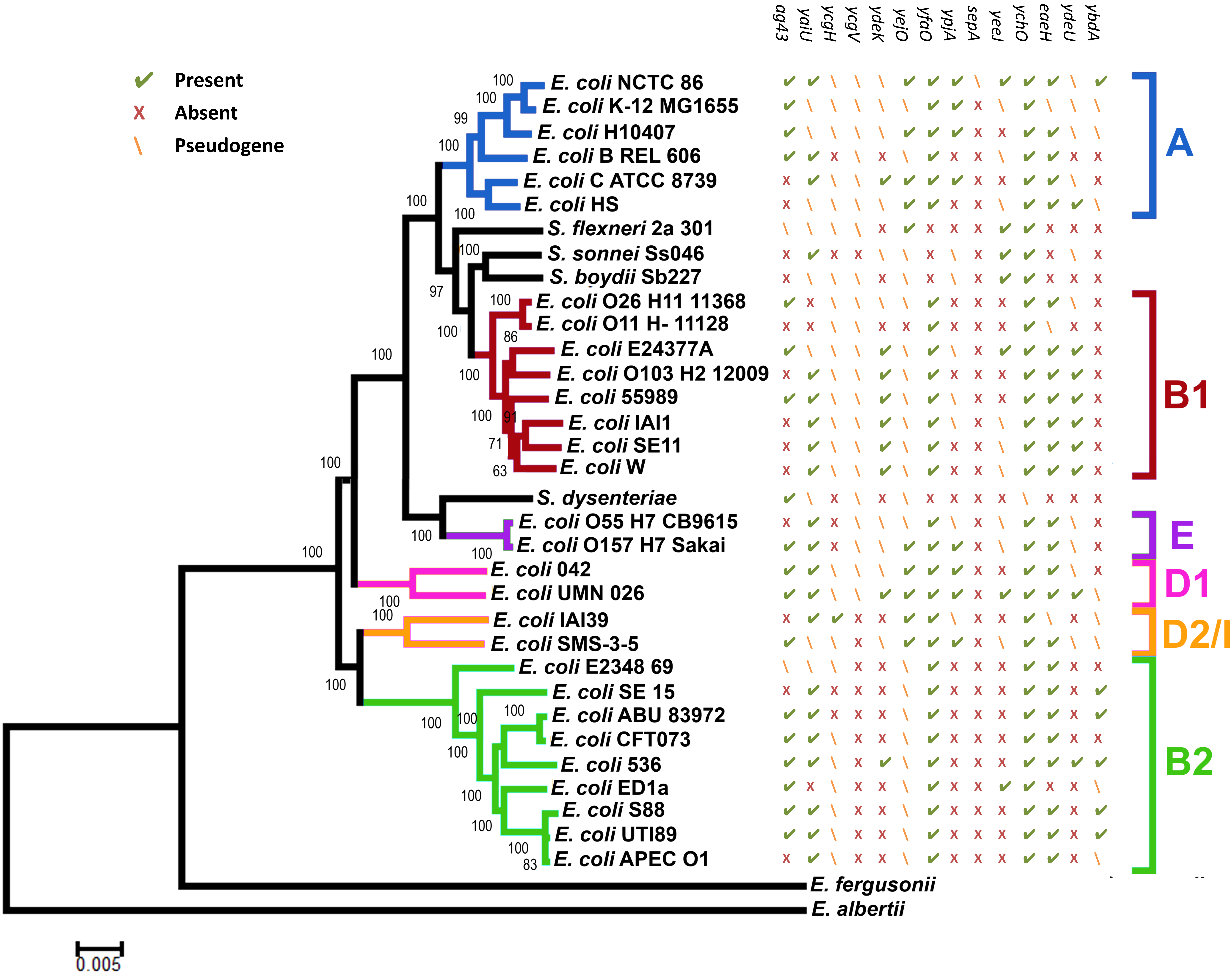

Present
Absent
Pseudogene
ag43
yaiU
ycgH
ycgV
ydeK
yejO
yfaO
ypjA
sepA
yeeJ
ychO
eaeH
ydeU
ybdA
E. coli NCTC 86
E. coli K-12 MG1655
E. coli H10407
E. coli B REL 606
E. coli C ATCC 8739
E. coli HS
S. flexneri 2a 301
S. sonnei Ss046
S. boydii Sb227
E. coli O26 H11 11368
E. coli O11 H- 11128
E. coli E24377A
E. coli O103 H2 12009
E. coli 55989
E. coli IAI1
E. coli SE11
E. coli W
S. dysenteriae
E. coli O55 H7 CB9615
E. coli O157 H7 Sakai
E. coli 042
E. coli UMN 026
E. coli IAI39
E. coli SMS-3-5
E. coli E2348 69
E. coli SE 15
E. coli ABU 83972
E. coli CFT073
E. coli 536
E. coli ED1a
E. coli S88
E. coli UTI89
E. coli APEC O1
E. fergusonii
E. albertii
A
B1
E
D1
D2/I
B2
0.005

Supplemental Figure 9: Type 6 Secretion System. Alignment *E. coli* NCTC 86 with other *E. coli* that possess the Type 6 secretion system. *E. coli* NCTC 86 has the strongest homology with *E. coli* 55989. CDSs are indicated by arrows coloured according to the GC content of the gene

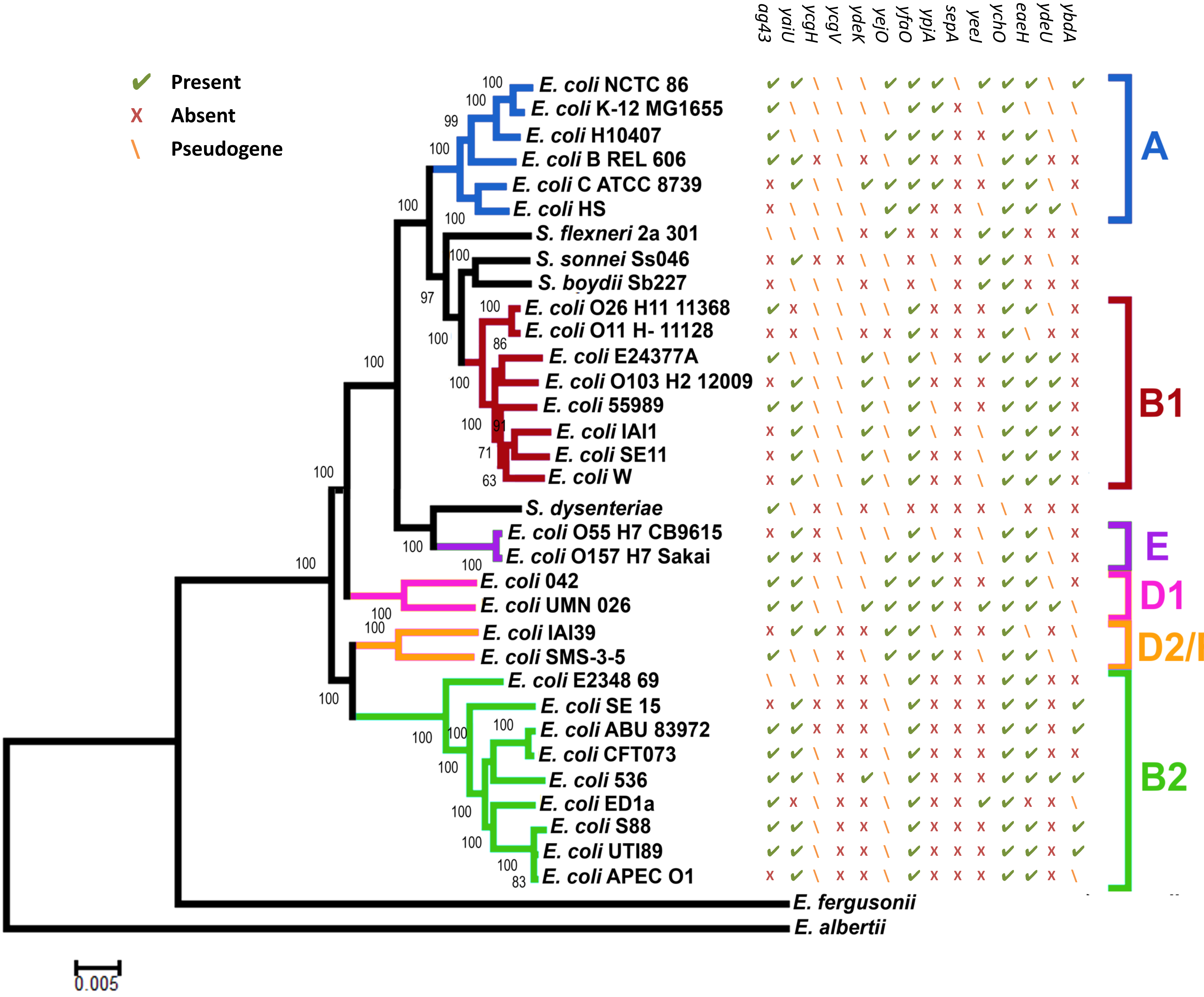

Present
Absent
Pseudogene
ag43
yaiU
ycgH
ycgV
ydeK
yejO
yfaO
ypjA
sepA
yeeJ
ychO
eaeH
ydeU
ybdA
E. coli NCTC 86
E. coli K-12 MG1655
E. coli H10407
E. coli B REL 606
E. coli C ATCC 8739
E. coli HS
S. flexneri 2a 301
S. sonnei Ss046
S. boydii Sb227
E. coli O26 H11 11368
E. coli O11 H- 11128
E. coli E24377A
E. coli O103 H2 12009
E. coli 55989
E. coli IAI1
E. coli SE11
E. coli W
S. dysenteriae
E. coli O55 H7 CB9615
E. coli O157 H7 Sakai
E. coli 042
E. coli UMN 026
E. coli IAI39
E. coli SMS-3-5
E. coli E2348 69
E. coli SE 15
E. coli ABU 83972
E. coli CFT073
E. coli 536
E. coli ED1a
E. coli S88
E. coli UTI89
E. coli APEC O1
E. fergusonii
E. albertii
A
B1
E
D1
D2/I
B2
0.005
